# Immunological distinctions between nonalcoholic steatohepatitis and hepatocellular carcinoma

**DOI:** 10.1038/s12276-020-0480-3

**Published:** 2020-08-07

**Authors:** Seo-Young Koo, Eun-Ji Park, Chang-Woo Lee

**Affiliations:** 1 Department of Molecular Cell Biology, Samsung Medical Center, Sungkyunkwan University School of Medicine, Suwon, 16419 Republic of Korea; 2 Department of Health Sciences and Technology, SAIHST, Sungkyunkwan University, Seoul, 06351 Republic of Korea

**Keywords:** Immunological disorders, Cell death and immune response

## Abstract

Nonalcoholic fatty liver disease (NAFLD), the most common cause of chronic liver disease, ranges from simple hepatic steatosis to nonalcoholic steatohepatitis (NASH), which is a more aggressive form characterized by hepatocyte injury, inflammation, and fibrosis. Increasing evidence suggests that NASH is a risk factor for hepatocellular carcinoma (HCC), which is the fifth most common cancer worldwide and the second most common cause of cancer-related death. Recent studies support a strong mechanistic link between the NASH microenvironment and HCC development. The liver has a large capacity to remove circulating pathogens and gut-derived microbial compounds. Thus, the liver is a central player in immunoregulation. Altered immune responses are tightly associated with the development of NASH and HCC. The objective of this study was to differentiate the roles of specific immune cell subsets in NASH and HCC pathogenesis.

## Liver disease: Immune cells in disease progression

Clarifying the role of specific cells in the immune system in the transition from non-alcoholic fatty liver disease (NAFLD) to liver cancer will help to understand disease progression and may open avenues towards new preventive and therapeutic strategies. NAFLD is the most common chronic liver disease. Growing evidence suggests that its most aggressive form, non-alcoholic steatohepatitis (NASH), can promote the development of liver cancer, the second most common cause of cancer deaths worldwide. Chang-Woo Lee and colleagues at Sungkyunkwan University, Suwon, South Korea review the immunological distinction between NASH and liver cancer, focusing on the levels and activities of six key types of immune system cells. Chronic inflammation mediated by the immune system can create conditions for NAFLD, NASH and liver cancer to develop and worsen.

## Introduction

Hepatocellular carcinoma (HCC) is the most common type of liver cancer and accounts for 70–85% of all liver cancer cases^1^. HCC is the sixth leading cause of cancer-related deaths globally and is expected to become the third leading cause of liver cancer-related deaths by 2030^2^. Such changes in HCC incidence are affected by obesity, type 2 diabetes, and nonalcoholic fatty liver disease (NAFLD), which is the most common liver disease^3^. Although NAFLD has a spectrum of liver pathologies similar to those of alcohol-induced fatty liver damage, NAFLD can occur in patients even in the absence of alcohol abuse^4^. NAFLD is characterized by a steatosis or the accumulation of triglycerides in lipid droplets inside hepatocytes (hepatic steatosis)^5^. Such accumulation of lipids is closely associated with metabolic syndromes such as obesity, type 2 diabetes, hypertension, and dyslipidemia^6^. NAFLD is highly prevalent on every continent. The global prevalence of NAFLD was ~25%. The Middle East has the highest prevalence rate of 32%, followed by South America (31%). Africa has the lowest prevalence at 14%^7^. NAFLD can progress to a more severe form called nonalcoholic steatohepatitis (NASH). NASH is marked by abnormal fat accumulation in the liver and immune cell infiltration into the liver due to chronic hepatitis and inflammation. In addition, it seems that most NASH patients develop progressive fibrosis^7^. NASH can cause liver diseases such as cirrhosis and HCC and is also associated with an increased risk of cardiovascular disease^8^.

The prevalence of NASH among NAFLD patients in the United States has been estimated to be 21% (95% confidence interval or CI: 19.85–22.95%). The prevalence of NASH in the United States accounts for ~3–4% of the entire population^9^. NASH is the fastest increasing cause of HCC in the United States^10^. As such, the incidences of NAFLD and NASH increase each year. Patients with these disorders are highly likely to have more than one metabolic syndrome. These individuals are at high risk of developing HCC^11,12^. The incidence of NAFLD/NASH-released HCC has continuously increased in many ethnic groups, including in the United States^13^ Europe^14–16^, South Korea^17^, and Japan^18^, over the past decades. A study released in 2010 stated that NAFLD/NASH (59%) was the most common etiological risk factor in the United States, followed by diabetes (36%) and hepatitis C virus (22%)^19^. Given recent advances in anti-hepatitis C virus (HCV) therapy, NASH is highly likely to become a major cause of progressive liver disease within the next three decades.

Thus, the epidemiology of NASH-associated HCC is continuously changing as the number of patients with metabolic syndrome surges yearly. Compared to patients with other causative factors, patients with NASH-associated HCC are more prone to complications such as diabetes, obesity, dyslipidemia, and hypertension. These factors can exacerbate the clinical complexity of patients and eventually result in a difficult situation for clinical management. Additionally, although patients with lesions caused by HCV or HBV can be partially treated because of the development of treatments, effective treatment is currently unavailable for NASH-associated HCC patients^20^. To overcome this growing burden of NASH and NAFLD/NASH-HCC, it is crucial to understand the factors associated with NASH and HCC to develop preventive and therapeutic strategies.

## Importance of the microenvironment during NASH and HCC pathogenesis

Recent studies have shown that the liver microenvironment may play a crucial role in NAFLD/NASH and HCC progression. The liver provides a unique proinflammatory microenvironment that is composed of a variety of immunologically active cells, including Kupffer cells (KCs), T cells, antigen-presenting cells (APCs), and hepatic stellate cells (HSCs)^21,22^. In pathological liver injury, these cells are part of a complex proinflammatory and fibrogenic background, and hepatocyte death occurs, promoting disease progression. Various pathobiological factors, including proinflammatory cytokines (such as interleukin (IL)-6 and tumor necrosis factor (TNF)-α), leptin, hyperinsulinemia, the gut microbiota, bile acid, and free fatty acid, can interact with components in the liver microenvironment. These factors may cause inflammation, fibrosis, and lipotoxicity as a result of interactions with the liver microenvironment. In the long term, the interactions of these factors with the liver microenvironment may lead to the progression to NASH and increase the possibility of HCC development^21^.

A proinflammatory microenvironment created by toxic lipid-induced hepatocyte injury (lipotoxicity), which may occur under NASH conditions, has a significant effect on the deterioration of NASH^23^. NASH may trigger the death of liver cells by inducing metabolic stress in hepatocytes. The generation of damage-associated molecular patterns (DAMPs) occurs after an influx of activated immune cells and is called sterile chronic inflammation. HCC is an inflammation-related cancer. A chronic inflammatory state can trigger the initiation and development of cancer^24^. Altered immune responses in NASH with chronic inflammation are also associated with the development of HCC^25–27^.

Various factors and microenvironmental changes have been reported in HCC patients. In particular, cancer-related microenvironmental components such as immune cells, fibroblasts, endothelial cells, and extracellular matrix (ECM) can facilitate tumor cell differentiation, proliferation, and invasion^26,28^. Thus, it is crucial to understand that the microenvironment of the liver plays a key role in the pathogenesis of NAFLD, NASH, and HCC. The pathogenesis of NASH and HCC is affected by various factors within the microenvironment. However, as inflammation is the most prominent feature of NASH and HCC, the present study reviews the function of immune cells in the respective microenvironments of NASH and HCC.

## Immunological differences in the microenvironment associated with NASH and HCC progression

### NK cells

**Fig. 1:**
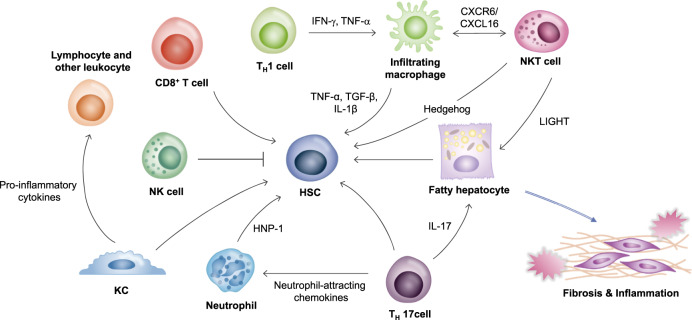
The immunological microenvironment of nonalcoholic steatohepatitis. The liver has the largest population of resident macrophages (KCs) and high densities of NK cells, NKT cells, various T cells and liver-transiting and/or resident lymphocytes, with a higher ratio of CD8^+^ T cells to CD4^+^ T cells than that in the periphery. Liver nonparenchymal cells are associated with innate and adaptive immune systems and control the liver microenvironment. During NASH progression, these cells functionally interact with each other by secreting cytokines and chemokines to induce profibrotic and proinflammatory signals. Immune cells also interact with other cells in the liver microenvironment, especially hepatic stellate cells (HSCs) and hepatocytes, to promote fibrosis and inflammation that can affect disease severity. Th1 cells can affect macrophages by secreting IFN-γ and TNF-α. These macrophages can produce cytokines to activate HSCs. CD8^+^ cells, Th17 cells, KCs, and neutrophils also contribute to the activation of HSCs. In contrast, NK cells inhibit the activation of HSCs. NKT cells and Th17 cells induce lipid accumulation in hepatocytes. Thus, altered immune regulation in the liver microenvironment can eventually lead to NASH development.

Natural killer (NK) cells are a group of innate immune cells that show cytolytic activity against cells under stress, such as tumor cells and virus-infected cells^29^. NK cells play crucial roles in NAFLD and NASH (Fig. 1). The number of NK cells is increased in hepatic inflammation associated with NASH^30^. The number of ligands of various NK cells is increased in the liver^31^. NK cells play an antifibrotic role and prevent the development of liver fibrosis by killing HSCs that cause fibrosis^31^. The cytolytic activity of NK cells is increased either by upregulating stress ligand activity or by downregulating HSC inhibitory ligands. However, HSCs that fail to undergo senescence can resist NK cell-mediated killing, while senescent HSCs decrease fibrogenesis and increase the inflammatory response. HSCs are chronically activated in NASH due to dysregulated senescence^32^. HSCs can resist the cytolytic function of NK cells in NASH. Additionally, NK cell activation by inflammatory cytokines such as IL-12, IL-15, and IL-18 contributes to the production of interferon (IFN)-γ and the formation of a proinflammatory environment^33–35^.

**Fig. 2:**
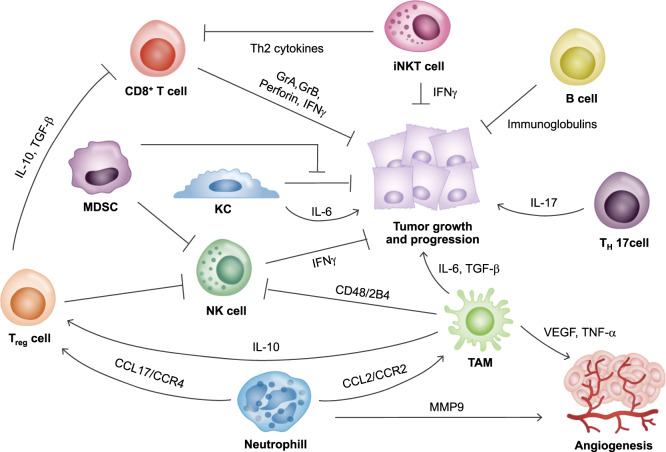
The immunological microenvironment of hepatocellular carcinoma. In the HCC liver microenvironment, a large population of immune cells, Th17 cells, Tregs, iNKT cells, TAMs, and neutrophils are activated. However, immune cells with antitumor functions such as NK cells, CD8^+^ T cells, and KCs are mostly defective. The number of NK cells is decreased, and their function is suppressed by Tregs and MDSCs. KCs have dual roles in the tumor microenvironment. These cells secrete IL-6 to promote tumor development. KCs also have antitumor functions. However, the antitumor function of KCs is suppressed by MDSCs. CD8^+^ T cells can directly inhibit tumor growth by secreting granular components and cytokines. However, these cells are suppressed by Tregs in the HCC microenvironment. TAMs play various roles during HCC progression, including affecting angiogenesis. Neutrophils can also induce angiogenesis while attracting protumorigenic cells, such as TAMs and Tregs. These immune cells play various roles and can exacerbate HCC development by supporting tumor growth, progression, and angiogenesis.

As mentioned above, NK cells usually perform antitumor functions through surveillance. The surveillance of NK cells in tumors is crucial in combating HCC (Fig. 2). However, in HCC conditions, the number of NK cells is reduced. The production of IFN-γ and cytotoxic functions are also impaired. This is due to increased Tregs in HCC patients^36^. In addition, it seems that NK cell function is decreased by myeloid-derived suppressor cells (MDSCs) during the development of HCC (Fig. 2). Thus, NK cells can exert antitumor effects. However, in the case of HCC, these cells are defective because they exist in an ineffective form, which is unable to remove tumor cells^37^.

### T lymphocytes

T cells play a regulatory role by controlling cells that are involved in immune responses, including B cells and T cells. There are two subsets of T cells: CD4^+^ helper T (Th) cells and CD8^+^ cytotoxic T cells. Th cells can be classified into Th1 and Th2 cells. Th1 cells produce IL-2 and IFN-γ and are involved in cellular immunity by regulating macrophages (Fig. 1). Th2 cells produce IL-4, IL-5, and IL-13 and are involved in humoral immunity by regulating eosinophils^38^. Another subset of Th cells is Th17 cells that secrete IL-17. Th17 cells are involved in the progression of many autoimmune diseases and inflammatory disorders. Th17 cells also secrete IL-22 and granulocyte-macrophage colony-stimulating factor (GM-CSF) to regulate neutrophils^39^ (Fig. 1). CD8^+^ cytotoxic T cells express clonally distributed receptors for foreign antigens, undergo marked proliferation and production of IFN-γ in response to infection, and kill any cell that expresses target antigens as an integral component of immunological memory^40,41^ (Fig. 1). The importance of these T cells in NAFLD has been suggested. It has been shown that T cell-deficient mice do not develop steatosis, hepatic inflammation or fructose-induced NAFLD^42^.

The selective loss of CD4^+^ T cells occurs in NAFLD. NAFLD induced by a methionine/choline-deficient (MCD) diet can trigger the loss of CD4+T lymphocytes and promote the development of HCC in liver-specific MYC oncogenic transgenic mice. In the same context, obesity-induced lipid accumulation can result in the selective loss of CD4^+^ T lymphocytes and promote disease progression from NAFLD to hepatocellular carcinoma^43^. These CD4^+^ T cells are biased to Th1 and Th17 subtypes in NASH conditions^44^. Th1 cells can promote the differentiation of macrophages into M1 macrophages that play a proinflammatory role by secreting IFN-γ and TNF-α^45,46^. Th17 cells secrete IL-17, which exacerbates hepatic steatosis and inflammation and induces the transition from simple steatosis to hepatic steatosis^47,48^. Th17 cells also produce neutrophil-attracting chemokines to recruit neutrophils and lymphocytes in NASH^49^. Fibrosis progression is further induced through HSCs^50^. In contrast, the number of hepatic regulatory T cell (Tregs) is decreased in NASH^48,51^. In mice with HFD-induced steatosis, Treg apoptosis can be induced by increased oxidative stress, whereas liver inflammation is markedly reduced by adoptive transfer of Tregs^52^. In fact, the ratio of Th17 cells/Tregs is higher in NASH patients than in NAFLD patients and normal controls, indicating that the ratio of Th17/Tregs may play a crucial role in the NASH environment^53^.

The number of Th17 cells and the level of IL-17 are increased in tumor tissues of patients with HCC (Fig. 2). These increases are associated with low survival and increased postoperative recurrence, indicating that Th17 cells may accelerate HCC progression. Systemic IL-17A causes infiltration of neutrophils into adipose tissue, which generates a feed-forward mechanism that exacerbates NASH by releasing fatty acids and inducing DNA damage in hepatocytes, thus promoting HCC^54^. The number of Tregs is significantly increased in HCC patients^55^. An increase in Tregs is mainly observed around tumor sites. Tregs extracted from the tumor site contribute to uncontrolled growth of HCC cells by inhibiting the proliferation of CD8^+^ cytotoxic T cells (Fig. 2), leading to poor clinical outcome and poor survival of HCC patients^56^.

The infiltration of CD8^+^ cells, another T cell subset, occurs in the context of NASH^57^. The recruitment of hepatic CD8+ T cells is increased NF-κB1-knockout NASH model mice. This increase in hepatic CD8^+^ T cells exacerbates liver inflammation and fibrosis in NF-κB1-knockout mice, indicating that CD8^+^ T cells are involved in NASH progression and fibrosis^58^. The depletion of CD8^+^ T cells could halt the progression of NASH and decrease fibrosis^57^. CD8^+^ T cell activation is involved in the development of not only NASH but is also subsequent liver cancer in mice fed a choline-deficient high-fat diet. In the transition from NASH to HCC, the activation of the NF-κB signaling pathway in hepatocytes is induced by CD8^+^ T cells via crosstalk with hepatocytes^59^.

CD8^+^ T cells are abundant in the early stages of HCC. Their number decreases as the disease progresses. CD8^+^ cells secrete granule components such as perforin and granzyme B (GrB) and play a cytolytic role (Fig. 2). These cells also possess antitumor functions. However, since these activities are significantly reduced and most of these cells exist in a quiescent state, the expression of cytolytic molecules in CD8^+^ T cells in HCC patients is markedly low. It appears that impaired CD8^+^ T cells can produce and release granzyme A (GrA), and GrB production might be interrupted by the increased numbers of Tregs in tumors^60^ (Fig. 2). Furthermore, the ability of CD8^+^ T cells to produce IFN-γ is impaired^61^.

### NKT cells

Natural killer T (NKT) cells are unconventional cells that express NK and T cell surface markers and secrete various cytokines, such as IFN-γ and IL-4, to control innate and adaptive immunity. NKT cells account for 30% of hepatic nonparenchymal cells^62,63^. NKT cells can produce factors that modulate inflammatory and fibrogenic responses in the liver^64^ (Fig. 1). The numbers of NKT cells and CD8^+^ T cells are increased in NASH patients^59^. This accumulation of hepatic NKT cells is also observed in the MCD diet-induced NASH model. Activation of the hedgehog pathway occurs during NASH-related fibrogenesis, leading to the induction of factors involved in the recruitment, retention, and viability of NKT cells. Accumulated NKT cells can stimulate HSCs to become myofibroblasts and promote the progression of NASH-associated fibrosis^64^ (Fig. 1). Additionally, an increase in CXCR6 expression in NKT cells and subsequent binding to CXCL16, which is expressed in endothelial cells and macrophages, in NASH can lead to the accumulation of NKT cells that produce proinflammatory cytokines such as IL-4, IFN-γ, and TNF-α, thus inducing inflammation via macrophage activation^65^ (Fig. 1). Moreover, hepatic NKT cells can secrete LIGHT to promote hepatic steatosis and liver damage through inflammatory cytokines and CD8^+^ T cells, contributing to canonical NF-κB signaling and stimulating the transition from NASH to HCC^59^.

There are more NKT cells in tumors than in blood in HCC patients^66^. Based on differences in T cell receptor (TCR) usage, these NKT cells can be classified into two types: Type I and Type II. Type I NKT cells express invariant TCRα chains that are readily detectable by α-galactosylceramide (α-GalCer)-loaded CD1d tetramers, while type II NKT cells express a broader TCR repertoire^67,68^. However, studies on Type II NKT cells in the liver are insufficient because of the absence of a specific marker. Thus, Type I NKT cells called invariant NKT (iNKT) cells have been mostly studied in patient tumors (Fig. 2). iNKT cells perform dual roles. These cells either promote the antitumor response by activating effector T cells or promote tumor growth by inducing the Th2 response through the recruitment of Tregs (Fig. 2). CD4^+^ and CD4^−^ iNKT cell subsets both produce Th1-type cytokines such as IFN-γ. However, CD4^+^ subsets show enhanced secretion of Th2 cytokines and regulatory activity. In the tumor microenvironment, the number of CD4-expressing iNKT cells is increased in HCC tumors, thus producing more Th2 cytokines, preventing the expansion of tumor Ag-specific CD8^+^ T cells, and promoting tumor growth^69,70^. On the other hand, iNKT cells exert antitumor effects by inhibiting the inflammatory response through oncogenic β-catenin^71^.

### Macrophages

Macrophages that circulate in the blood or exist in various organs and tissues are the first barriers to any disease. These cells play the most important role in innate and acquired immunity. Macrophages are generally divided into two phenotypes: classically activated (M1) macrophages and alternatively activated (M2) macrophages^72^. M1 macrophages are characterized by an IL-12^hi^IL-23^hi^IL-10^lo^ phenotype. These cells produce toxic effector molecules (ROS and NO) and inflammatory cytokines such as IL-1β, TNF, and IL-6. They participate in Th1 responses and mediate resistance to intracellular parasites and tumors. In contrast, M2 cells are characterized by an IL-12^lo^IL-23^lo^IL-10^hi^TGF-β^hi^ phenotype *in vitro*. M2 cells participate in polarized Th2 responses, allergies, parasite clearance, dampening of inflammation, tissue remodeling, angiogenesis, immunoregulation, and tumor promotion^73^. In the liver, hepatic resident macrophages are called KCs and are involved in inflammation signaling and metabolic changes.

In NASH, various metabolic syndromes and insulin resistance promote the accumulation of free fatty acids (FFAs) in hepatocytes and blood, triggering the innate immune response by stimulating lipotoxin and LPS. In a healthy liver, the KCs primarily function as the body’s frontline defense against phagocytosis and pathogens from the portal vein and arterial circulation^74^. The level of KCs decreases in NASH, while the infiltration of Ly6C^+^ monocyte-derived macrophages is increased in the early stage of NASH in an MCD diet model^75^. In NASH, toll-like receptor (TLR) 4 in KCs is more highly expressed than other TLRs^76^. When LPS binds to TLR4, KCs are activated, and the production of proinflammatory cytokines such as TNF-α, IL-1β, IL-2, IL-6, IL-10, and IFN-γ is enhanced, triggering the recruitment of lymphocytes and other leukocytes^77^ and promoting the activation of NF-κB, MAPK, ERK1, p38, JNK, and IRF3^78^. Additionally, the expression of hepatic inflammatory genes is increased by oxidized low-density lipoprotein (LDL) trapped in KC lysosomes in a NASH model^79^. Recent studies have shown an increase in endolysosomal lipid accumulation in KCs during the progression from NAFLD to NASH^80^, indicating that KCs play a crucial role in the progression of NASH. Along with KCs, infiltrated Ly6C^+^ macrophages produce cytokines such as TNF-α and IL-1β, thus promoting inflammation and activating HSCs^81^.

KCs act as a major mediator in fibrosis (Fig. 1). In vivo KC depletion inhibits HSC activation and fibrosis development in a mouse model of fibrosis^82^. Indeed, KCs can secrete profibrogenic cytokines such as transforming growth factor-β (TGF-β) and platelet-derived growth factor (PDGF)^78^. In other words, KCs contribute to inflammation and fibrosis progression through various processes, exacerbating NASH (Fig. 1). In addition, KCs can recruit CD4 T cells and increase T cell tolerance^83^. In the late stage of NASH, the release of DAMPs such as intracellular DNA from dying hepatocytes may trigger the activation of KCs. It has been reported that hypoxia in the liver may activate KCs by upregulating transcription factors such as hypoxia inducible factor 1α (HIF-1α)^84^.

KCs contribute to immune surveillance and play a role in antitumor immunity. However, KCs present in the tumor microenvironment (TME) of HCC have low expression of costimulatory molecules but high expression of coinhibitory molecules. Moreover, due to the presence of MDSCs, the antitumor function of KCs is suppressed^85^ (Fig. 2). Recently, it has been proven that KCs are involved in the development of HCC^22^. In a diethylnitrosamine (DEN)-induced HCC model, hepatocellular carcinogenesis was attenuated when the activation of KCs was mitigated by deletion of the proinflammatory myeloid cell surface receptor triggering receptor expressed on myeloid cell-1 (TREM-1), which is expressed by KCs. It has been revealed that the activation of KCs is crucial for tumor development in the early stage of chemical-induced carcinogenesis^86^. Furthermore, IL-1a secreted either by oncogene-induced senescent hepatocytes or by apoptotic hepatocytes in the DEN model can induce the production of IL-6 in KCs and trigger compensatory proliferation and tumor progression, which are essential for HCC development^22^. Once a primary tumor is established, liver-infiltrated macrophages play a more prominent role than KCs. Macrophages infiltrate near tumors. Macrophages present in the TME are called tumor-associated macrophages (TAMs)^87^. A tumor with highly infiltrated TAMs leads to a worse prognosis in HCC^88^ (Fig. 2). TAMs are attracted by chemokines, such as macrophage colony-stimulating factors (M-CSF), produced by tumor cells. These cells infiltrate the tumor site^89^. Additionally, glypican-3, which is expressed in HCC, can recruit macrophages^90^.

In the HCC microenvironment, the transformation of macrophages into the M2 phenotype is further amplified by factors such as CSF1, Wnt, HIFs, IL-8, and HMGB1^91^. Macrophages that are transformed into the M2 phenotype can exacerbate HCC. M2-polarized macrophages enable epithelial–mesenchymal transition (EMT) and the migration of HCC cells via the TLR4/signal transducer and activator of transcription (STAT) 3 signaling pathway^92^. Moreover, increased stability of HIF-1α in hypoxic conditions in HCC increases the secretion of IL-1β and promotes EMT and metastasis of tumor cells^93^. TAMs can also secrete IL-6 and promote the expansion of cancer stem cells and tumor formation through IL-6/STAT3 signaling^94^ (Fig. 2). Additionally, TAMs can secrete a larger amount of TGF-β than other macrophages and facilitate the progression of tumors by obtaining cancer stem cell-like properties through TGF-β1-induced EMT^95^. Indeed, the level of TGF-β is increased in HCC patients^96^.

TAMs can interact with various immune cells. Macrophages can trigger the accumulation of Tregs through the secretion of IL-10 and inhibit the activation of other CD4^+^CD25^−^ cells to induce the progression of HCC^97^. Macrophages can also promote the expansion of Th17 cells and tumor growth by secreting various proinflammatory cytokines^98^, causing dysfunction and apoptosis of NK cells through interactions with NK cells via CD48/2B4^99^. In addition to the immune response, TAMs contribute to angiogenesis, which exacerbates cancer. TAMs can produce proangiogenic factors such as VEGF and TNF-α, interact with the ECM, promote angiogenesis, and facilitate tumor cell invasion^87^ (Fig. 2).

### B cells

B cells are immune cells that play a role in antibody secretion, antigen presentation, T cell costimulation, and cytokine secretion. B cells play an immunomodulatory role and contribute to autoimmunity and disease pathogenesis^100^. Relatively fewer studies have been conducted on B cells than on other kinds of immune cells in the NASH microenvironment. However, the role of B cells in NASH is gradually being elucidated. B cell-activating factor (BAFF), a TNF superfamily member, is secreted by adipocytes. BAFF regulates the maturation and development of B cells^101^. NASH patients have higher levels of BAFF than control patients with simple steatosis. BAFF is associated with the degree of ballooning hepatocytes and hepatic fibrosis^102^. In BAFF-knockout mice, the number of mature B cells is decreased. Recent studies have shown that deletion of BAFF in the NAFLD model attenuates hepatic fat accumulation, inhibits inflammation and fibrosis in VAT, improves insulin resistance, and weakens liver steatosis. These results indicate that BAFF plays a role in exacerbating NAFLD and NASH^103^. BAFF-knockout mice show reduced systemic inflammation^104^. In response to LPS stimulation, hepatic B cells produce more IFN-γ, IL-6 and tumor necrosis factor (TNF)-α but less IL-10 than those from secondary lymphoid tissue, indicating that hepatic B cells promote inflammatory responses^105^. Increased TGF-β levels in NASH patients^106^ trigger the transformation of IgM-expressing B cells into IgA-expressing B cells with regulatory activity. The level of serum IgA is increased in NASH patients. A high level of IgA is associated with the state of fibrosis^107,108^. Additionally, the accumulation of liver-resident IgA^+^PDL1^+^ cells that secrete IL-10 concurrent in NASH can promote tumor growth by inhibiting the function of CD8^+^ T cells^107^.

In the development of HCC, B cells can inhibit the growth of established tumors, while T cells prevent the onset of tumor formation^109^. An increased level of tumor-infiltrating B cells can lead to better clinical outcomes. However, intratumor infiltration of B cells is impaired during the progression of HCC^110^. B cells secrete immunoglobulin with a direct antitumor effect that is beneficial for HCC patients^111^ (Fig. 2). When mature B cells are transplanted with hepatoma cells in B cell-depleted mice, tumor growth is not controlled, and the activation of local T cells is decreased, indicating that B cells might play a crucial role in cancer^112^. In contrast, it has been reported that the infiltration of CD20^+^ B cells is associated with poor survival in HCC patients. Ablation of CD20^+^ B cells induces senescence-mediated fibrosis resolution and promotes inhibition of the protumorigenic TNF-α/NF-κB pathway in Mdr2^−/−^ mice, which is an inflammation-associated cancer model^113^.

### Neutrophils

Neutrophils are innate immune cells that account for an overwhelming majority of circulating lymphocytes. Neutrophils serve as the frontline defense of the immune system. They are the first cells to infiltrate in the event of acute liver injury. Neutrophils can capture and destroy invasive microorganisms via phagocytosis, intracellular degradation, granule release, and the formation of neutrophil extracellular traps (NETs). These cells serve as mediators of inflammation^114^. NASH is characterized by the infiltration of a large number of neutrophils (Fig. 1). In the MCD diet-induced NASH model, when the infiltration of hepatic neutrophils is suppressed, an early stage of NASH is significantly attenuated, resulting in reduced levels of serum alanine transferase (ALT) and proinflammatory mRNA, indicating that neutrophils may exacerbate the early stage of NASH^115^. Neutrophil-derived myeloperoxidase (MPO) is also associated with hepatic cholesterol accumulation, inflammation, and fibrosis. MPO enhances macrophage cytotoxicity and induces neutrophil activation^116^. Additionally, neutrophil-derived human neutrophil peptide (HNP)-1 induces hepatic fibrosis through HSC proliferation and exacerbates NASH^117^ (Fig. 1). Indeed, the neutrophil-to-lymphocyte ratio (NLR) has been found to be high in NASH patients with advanced fibrosis, indicating that neutrophils are a key factor in fibrosis and NASH^118^.

Neutrophils also play a crucial role in the transition from NASH to HCC (Fig. 2). The levels of markers of NETs (large, extracellular, web-like structures composed of cytosolic and granule proteins that are assembled on a scaffold of decondensed chromatin^119^) expelled by neutrophils are increased in sera samples from NASH patients. NETs play a key role in the formation of chronic inflammatory liver microenvironment in NASH that promotes the progression to HCC. The formation of NETs has been observed in livers of STAM mice (NASH induced by neonatal streptozotocin and a high-fat diet), followed by an influx of monocyte-derived macrophages, the formation of inflammatory cytokines, and progression to HCC^120^.

Neutrophils are enriched mainly around the peritumoral stromal region in HCC. The frequency of neutrophil infiltration in the liver is an indicator of poor survival in HCC^121,122^. Increased levels of IL-17 in HCC promote the secretion of CXC chemokines to recruit neutrophils around the peritumoral stromal regions (Fig. 2). Accumulated neutrophils can promote the secretion of matrix metalloproteinase-9 to induce angiogenesis^122^. Additionally, CXCR6^high^ tumor cells in HCC can secrete cytokines such as IL-1β, IL-6, and IL-8, induce the infiltration of CD66b^+^ neutrophils, and promote neutrophil-mediated multiple protumor responses^123^. Tumor-associated neutrophils in HCC can promote tumor growth and progression by recruiting macrophages and Tregs, which are increased after sorafenib treatment to resist the antitumor effect of treatment^124^. Additionally, neutrophil infiltration in HCC can produce hepatocyte growth factors (HGFs) and promote the growth and metastasis of malignant cells that are activated by GM-CSF, which is secreted by hepatoma cells in the HCC microenvironment^125^. Taken together, these findings indicate that neutrophils play a protumorigenic role in HCC progression, growth, and metastasis.

### Other cell types

Other myeloid cells also have various roles in NASH and HCC pathogenesis. Particularly, dendritic cells (DCs) are the most powerful APCs and can induce adaptive immune responses and generate tolerance to self-antigens^126^. Since DCs induce the adaptive immune response, the importance of DCs during hepatic inflammation has emerged. The hepatic DC population becomes expanded and mature in the NASH liver^127^. When DCs are depleted, intrahepatic fibrosis-associated inflammation is markedly exacerbated. Hence, in NASH, DCs can limit CD8^+^ T cell expansion and restrict Toll-like receptor expression and cytokine production in innate immune effector cells, including KCs, neutrophils, and inflammatory monocytes^127^. DCs can capture HCC-related antigens, process antigens, and activate antigen-specific T cells to remove tumors. However, in advanced HCC, the functions of DCs and IL-12 production are impaired. Thus, antigen-specific T cells cannot be activated properly^128^.

Monocytes play a pivotal role in inflammation and metabolic stresses. The infiltration of monocytes is an important feature of NASH. CD11b^int^Ly6C^hi^ monocytes appear to be the predominant source of TNFα production at a later time point in MCD diet-induced disease, leading to NASH progression^129^. These infiltrated monocytes have potent proinflammatory activities. In addition, together with KC, infiltrated monocytes can propagate lipid accumulation induced by the MCD diet mainly through the production of TNFα^129^. In HCC patients, PD-L1+ monocytes are highly enriched in the peritumoral stroma. These monocytes are activated to suppress tumor-specific T cell immunity. In fact, high infiltration of these monocytes is associated with poor survival of HCC patients^130^.

## Therapeutic perspective and conclusion

Acute inflammation is a useful reaction to achieve tissue recovery by promoting regeneration. Conversely, chronic inflammation is maladaptive and provides an environment that is conducive to the development of NASH and HCC. Chronic injury triggers the secretion of significant amounts of proinflammatory molecules, including IL-1, IL-6, TNF-α, and lymphotoxin-β, that can facilitate HCC development^24^. As mentioned above, NAFLD and NASH are major causes of the increased prevalence of HCC. Additionally, NASH-related HCC mainly occurs in the context of cirrhosis. Although NASH-related HCC cases occur mainly in cirrhotic patients, an increasing number of HCC cases have been reported in NAFLD and NASH patients with little or no cirrhosis^131^. While many studies on the respective microenvironments of NASH and HCC have been conducted, such studies on changes in the progression from NASH to HCC are insufficient. Since various immune cells have different functions that can affect the development of NASH and HCC, it is critical to develop an experimental model to identify the transition from NASH to HCC.

NASH with advanced cirrhosis is currently the primary etiology for liver transplantation and is estimated to become the leading indication for liver transplantation^132^. Thus, patients with NASH are at increased risk of adverse liver-related outcomes, with the degree of fibrosis contributing most significantly to this increased risk. However, medications have not yet been approved by the Federal Drug Administration (FDA) or European Medicines Agency (EMA) for the treatment of NASH. Many agents are currently being studied in clinical trials Phase 2 clinical trials have been for the TLR4 antagonist JKB-121 (ClinicalTrials.gov identifier: NCT02442687), which targets cells expressing TLRs, such as KCs, and solithromycin (ClinicalTrials.gov identifier: NCT02510599), which inhibits TNF-α/CXCL8 production and MMP9 activity in monocytic cells. Obeticholic acid (OCA) is a derivative of the primary human bile acid chenodeoxycholic acid (CDCA) and functions as an agonist of farnesoid X receptor (FXR). There was a randomized placebo-controlled, phase IIb trial and a large phase III trial to evaluate the safety and efficacy of OCA for the treatment of NASH and fibrosis^133^. Elafibranor is an agent currently in a phase III trial for the treatment of NASH (ClinicalTrials.gov Identifier: NCT02704403). Elafibranor, a dual peroxisome proliferator-activated receptor (PPAR)-α/δ agonist^133^, and cenicriviroc (CVC), a dual CCR2/CCR5 chemokine receptor antagonist that has been shown to play key roles in hepatic inflammation and fibrosis^134^, are being investigated in current phase III clinical trials in patients with NASH and fibrosis, respectively (ClinicalTrials.gov Identifier: NCT03028740). Additionally, the immune checkpoint inhibitors nivolumab (ClinicalTrials.gov identifier: NCT02576509), pembrolizumab (ClinicalTrials.gov identifier: NCT03062358), and tremelimumab (ClinicalTrials.gov identifier: NCT03298451) are undergoing phase III clinical trials in patients with HCC.

Various mechanisms and microenvironmental changes are involved in the onset and progression of NASH and HCC, raising a fundamental question on whether one targeted treatment would be effective. Indeed, most of the abovementioned drug candidates did not show expected therapeutic efficacies in phase II or phase III clinical trials. Importantly, a combination therapy that uses more than two types of targeted drugs to provide additional and/or synergistic effects could be a more effective strategy for NASH and HCC treatment. For instance, antidiabetic drugs or antifibrotic drugs with an FXR agonist, a well-known drug for NASH, have shown notable effects in clinical trials^135^. Furthermore, instead of using only checkpoint inhibitors, combined therapies targeting immune cells involved in the progression of HCC or targeting cells participating in angiogenesis with checkpoint inhibitors are now presenting promising outcomes^136,137^. However, combinational therapy that controls steatosis, chronic inflammation, and fibrosis could be the most efficient therapeutic strategy for NASH treatment. In addition, overcoming the hypocellular hepatic microenvironment is the most important condition for inducing efficient drug effects. Thus, developing effective drug candidates that can reverse the functions of immune cells in the respective microenvironments of NASH and HCC is expected to become a novel therapeutic strategy.
